# DTW-MIC Coexpression Networks from Time-Course Data

**DOI:** 10.1371/journal.pone.0152648

**Published:** 2016-03-31

**Authors:** Samantha Riccadonna, Giuseppe Jurman, Roberto Visintainer, Michele Filosi, Cesare Furlanello

**Affiliations:** 1 Fondazione Bruno Kessler, Trento, Italy; 2 Research and Innovation Centre, Fondazione Edmund Mach, San Michele all’Adige, Italy; Leibniz-Institute for Farm Animal Biology (FBN), GERMANY

## Abstract

When modeling coexpression networks from high-throughput time course data, Pearson Correlation Coefficient (PCC) is one of the most effective and popular similarity functions. However, its reliability is limited since it cannot capture non-linear interactions and time shifts. Here we propose to overcome these two issues by employing a novel similarity function, Dynamic Time Warping Maximal Information Coefficient (DTW-MIC), combining a measure taking care of functional interactions of signals (MIC) and a measure identifying time lag (DTW). By using the Hamming-Ipsen-Mikhailov (HIM) metric to quantify network differences, the effectiveness of the DTW-MIC approach is demonstrated on a set of four synthetic and one transcriptomic datasets, also in comparison to TimeDelay ARACNE and Transfer Entropy.

## Introduction

Inferring a biological graph (*e.g.*, a Gene Regulatory Network) from high-throughput longitudinal measurements of its nodes is one of the critical challenges in computational biology, and several are the proposed solutions to this still unanswered question [1–3]. Although the problem is strongly non-linear, a simple but widespread solution such as the coexpression networks via correlation measures provides a good approximation [4–8], even outperforming more complex approaches [4, 9–11]. This follows from the observation that functionally related genes share similar expression patterns [12], implying that coexpression and functional relationships are correlated [13–15]. Pearson Correlation Coefficient (PCC) is the most used similarity measure [16–18], although alternative correlation functions can be also employed [19–21]. However, PCC lacks sensitivity in case of non-linear relations [22] and time shift between signals [23–25], thus the reliability of a coexpression network would benefit from a measure taking care of these characteristics.

Diverse inference methods have been proposed to include more flexibility and, more generally, to build networks that can capture higher order interactions better than coexpression. Recent reviews [7, 26–34] provide a map of the landscape on network inference to date, while comparison studies and competitions (notably the DREAM challenges series) have tried to detect the optimal solution. However, the widespread range of application of network inference prevents from individuating an elective algorithm, with each novel method claiming advantage in a particular context, often with overoptimistic results [35]. The integration of the predictions from individual methods has thus been proposed to complement advantages while limiting flaws [35–38], however with limited operability, due for instance to the early stage of the ensemble techniques in this area [39, 40] and to the heterogeneity of the challenged tasks [41]. In terms of mathematical techniques, there is a wide choice of approaches alternative to correlation, including mutual information (MI) evaluation, partial correlation estimation, Bayesian network, statistical physics approaches to dynamic systems. In particular, ARACNE [42] for MI and WGCNA [16, 17] for correlation (and the algorithms derived from these, see Methods section) stand as reference methods [4, 9, 28]. Similarity measures specifically targeted to longitudinal data have also entered the game [43], either as variation of existing general purpose methods, or just transferred from other domains such as signal processing, finance, climatology. Recent reviews on similarity functions for time series list a wide diversity of mathematical strategies, including *e.g.*, cross-correlation, Hidden Markov Models, edit distance, Newey–West estimator, spectral distances [44–48].

In particular, two measures proved to be quite effective in tackling the two issues of non-linearity and time-shift: the Maximal Information Coefficient (MIC) [49] and the Dynamic Time Warping (DTW) [50]. MIC is a MI-based association measure aimed at detecting functional (linear and non-linear) dependencies between two variables [49, 51–53], part of the family of Maximal Information-based Nonparametric Exploration statistics. DTW is a classical measure evaluating the distance between two temporal sequences possibly varying in time or speed, applied to temporal sequences of video, audio, graphics and omics data [50, 54–57]. Operatively, DTW works by detecting the optimal mapping between the sequences via Dynamic Programming, to obtain an alignment; by backtracking, DTW provides a natural geometric representation of the time shift between two sequences.

As an alternative to PCC, here we propose to infer coexpression networks from omics time series data by a similarity measure integrating MIC with DTW. We define the DTW-MIC function as the root mean square of MIC and the similarity measure naturally induced by DTW. We quantify the performance of the DTW-MIC approach within a differential network framework based on the Hamming-Ipsen-Mikhailov (HIM) distance [58, 59], thus obtaining a quantification of the difference between the inferred network and the true network, whenever the true reference network is available. In particular, we evaluate DTW-MIC on four synthetic datasets generated by GeneNetWeaver (GNW) [60] following the guidelines of the DREAM Challenge [61]. Further, we apply the method on a gene expression reference dataset on T-cell activation in response to phorbol 12-myristate 13-acetate (PMA) and ionomycin treatment [62], showing a consistent improvement over PCC in network reconstruction. In addition to comparing DTW-MIC with the baseline reference PCC in the WGCNA framework, we evaluate the novel approach with respect to two algorithms differently estimating MI. We consider Transfer Entropy [63, 64], where the MI is integrated by the dynamics of information transport, and TimeDelay ARACNE [65]. The latter assumes that the underlying probabilistic model of the expression profiles is a stationary Random Markov Field and it outperforms the original ARACNE on longitudinal data. On average, the nets inferred by DTW-MIC were closer to the true network than the graphs inferred by both Transfer Entropy and TimeDelay ARACNE.

As a bioinformatics resource, we provide an implementation of the DTW-MIC measure, other association and inference functions, and the HIM distance within ReNette, the Open Source web framework for differential network analysis [66]. ReNette and its companion R package *nettools* [58] are available on the CRAN archive (http://cran.r-project.org) and on the GitHub repository https://github.com/MPBA/nettools.git.

## Methods

### Time series similarity measures

#### Maximal Information Coefficient

The Maximal Information Coefficient (MIC) measure is a member of the Maximal Information-based Nonparametric Exploration (MINE) family of statistics, introduced for the exploration of two-variable relationships in multidimensional data sets [49, 51, 53]. Operatively, the MIC value is obtained by building several grids at different resolutions on the scatterplot of the two variables, then computing the largest possible MI achievable over all grids and finally normalizing to the [0, 1] range, where larger values correspond to higher similarity. The two distinctive features of MIC are generality, *i.e.*, the ability of capturing variable relationships of different nature, and equitability, that is the property of penalizing similar levels of noise in the same way, regardless of the nature of the relation between variables.

Since its introduction in 2011, a debate arose in the scientific community regarding statistical flaws of MINE [67–75], such as tendency to overestimate MI and to generate false positives: superiority over MIC has also been claimed for alternative measures, such as Brownian distance correlation [76] and biweight midcorrelation [9]. Alternative statistics stemmed from the MIC definition such as Copula Correlation [77], GeneralizedMIC [78], Multivariate Maximal Correlation Analysis [79], Mutual Information based Dependence Index [80] and ImprovedAlgorithmMIC [81]. Altogether they do not match the popularity gained by the original MIC statistic, also in the computational biology community, *e.g.*, in the analysis and inference of various kinds of biological networks. MIC has been coupled to the Context Likelihood of Relatedness (CLR) [82] for network inference from steady state data [83, 84]; MIC has been used for the same purpose in integration with the Interaction Component Model [85]. MIC has been used as an association measure for omics and other data in several systems biology studies, for a partial list, see [86–97]; several studies have specifically considered control of false positive ratio [98–100]. MIC (and the other MINE statistics) can be computed in R [101] by using the *minerva* package [52].

**Fig 1:**
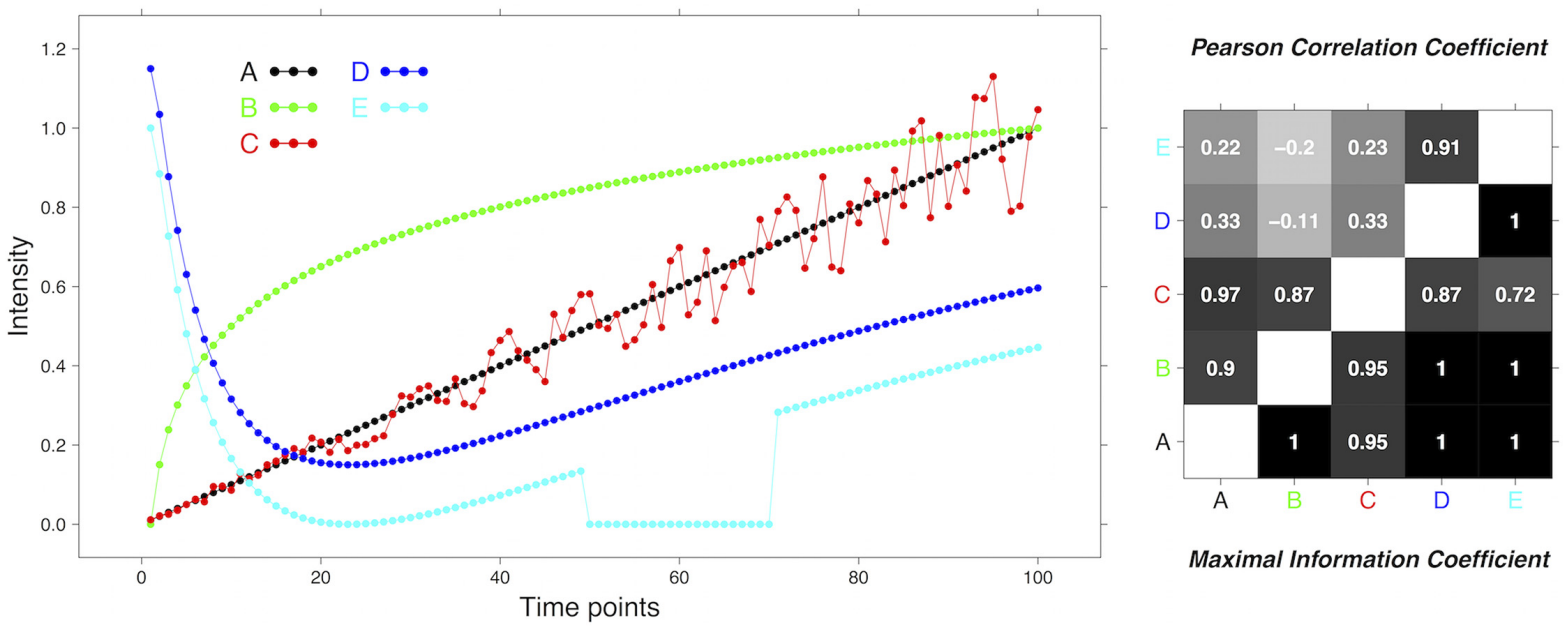
Example $E_{1}$. PCC versus MIC in a synthetic example with five time series *A*–*E* on 100 time points (left) and the corresponding PCC values (right panel, top-left triangle) and MIC values (right panel, bottom-left triangle) for all pairs of time series.

**Example $E_{1}$** To illustrate the difference between PCC and MIC in detecting non-linear relationships between two variables, we introduce a simple synthetic example $E_{1}$. Consider the following five time series with 100 time points {*t*_*i*_ = *i* : 1 ≤ *i* ≤ 100}:

$$\begin{array}{ccc} A(i) & = & 0.01i\\ B(i) & = & \log_{100}i\\ C(i) & = & 0.01i+\varepsilon (0.002i),\;\varepsilon (z)\in U(-z,z)\\ D(i) & = & 0.5\cos\log i+0.65\\ E(i) & = & \left\{ \begin{array}{cc} 0 & \text{for}\;50\leq i\leq 70\\ D(i)-0.15 & \text{otherwise}, \end{array}\right. \end{array}$$

where U(a,b) is the uniform distribution with extremes *a* < *b*. While *A*(*i*) is just 1/100–th of the identity map, *B*(*i*) is a logarithmic map, *C*(*i*) is obtained from *A*(*i*) by adding a 20% level of uniform noise, *D*(*i*) is a more complex non-linear map merging a trigonometric and a logarithmic relation and, finally, *E*(*i*) is obtained from *D*(*i*) by a vertical offset and then flattening to zero all the values in the time interval [50, 70]. In Fig 1 the plot of the five time series *A*–*E* is displayed together with the PCC and MIC values for all pairs of sequences. MIC is able to capture the functional relationship linking all pairs of time series, even in presence of a moderate level of noise: all MIC values are larger than 0.72, and in six cases out of ten MIC attains the upper bound 1. On the other hand, PCC is close to one only when evaluating the pairs (*A*, *B*), (*A*, *C*), (*B*, *C*) and (*D*, *E*), while all the remaining six cases display a correlation score smaller than 0.33, confirming that PCC is ineffective as a similarity measure for complex longitudinal data. As a relevant example, note that *B*(*i*) has a strong functional dependence from *D*(*i*) and *E*(*i*) although the shape of the corresponding curves are hugely different: this non-linear behaviour is well captured by MIC, with similarity value 1 to both (*B*, *D*) and (*B*, *E*), while the corresponding values for PCC are negative.

#### Dynamic Time Warping

Dynamic Time Warping (DTW) [54, 55] is a measure of distance between two sequences that takes care of time shifts. The DTW algorithm finds an optimal alignment between the two series by a non-linear warping of the time axes, also providing a measure of their dissimilarity. By construction, the similarity between curve shapes is a more important factor in DTW rather than the pointwise distance between the time series values. For a comprehensive reference, the reader is referred to [102].

Several variations to the original DTW algorithm have been proposed, first to overcome technical drawbacks and then to target specific data structures. Within the most important alternatives, we list DerivativeDTW [103], IterativeDTW [104], FastDTW [105], WeightedDTW [106], OnlineDTW [107], NearestNeighborDTW [108] and ComplexityInvariantDTW [109], possibily the most promising. Although the DTW alternatives often perform better in specific cases, for the original DTW robustness [106, 107] and effectiveness in a general scenario [110–112] are acknowledged. In particular, alternative measures to DTW specifically tailored to network inference do exist [113], but they have not been as extensively tested as DTW.

To obtain a similarity measure DTW_*s*_ from the distance DTW we use the function DTW_*s*_ = 1/(1 + DTW_*d*_), where DTW_*d*_ is the normalized distance between two series, as computed in the R package *dtw* [114].

**Fig 2:**
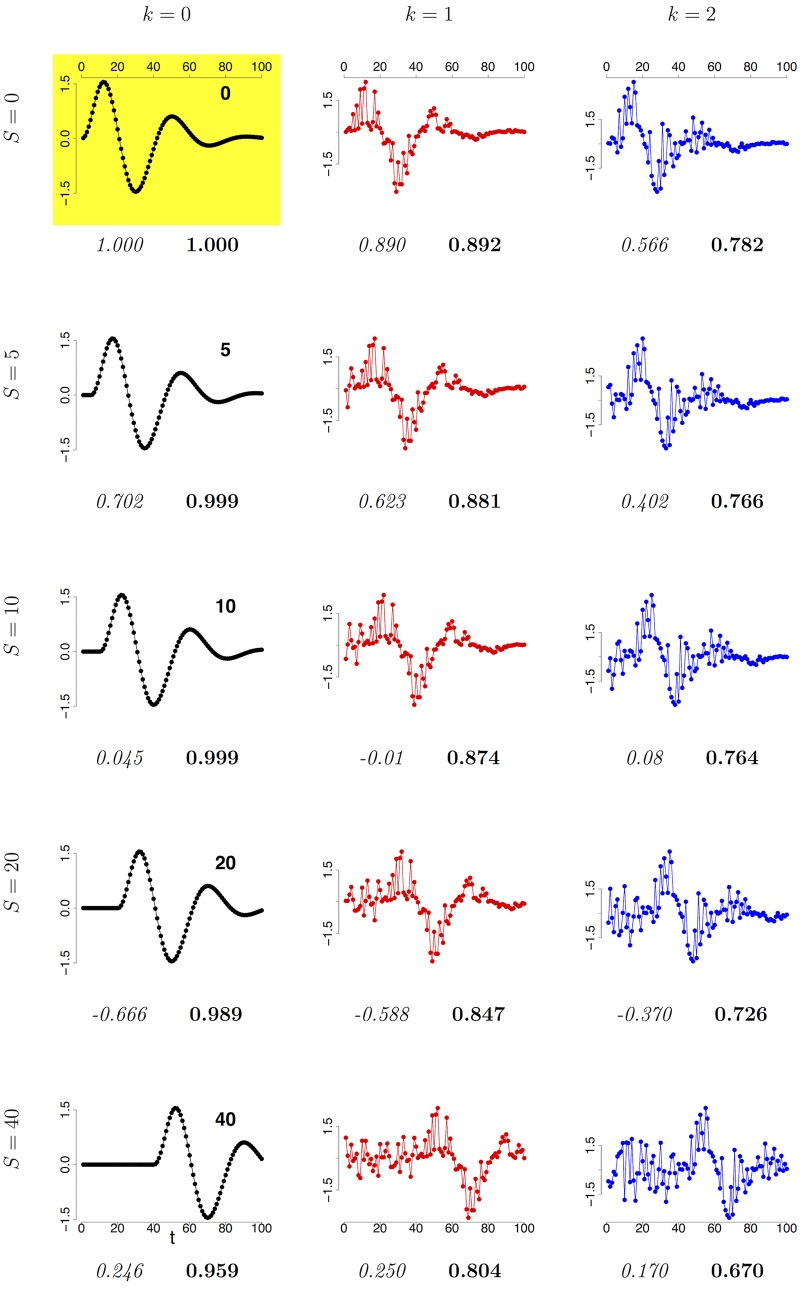
Example $E_{2}$. PCC and DTW_*s*_ versus the reference series *r* for the 15 time series $r_{s}^{\left[k\right]}\left(i\right)$ with *s* = 0, 5, 10, 20, 40 and *k* = 0, 1, 2. Each row corresponds to a different value of *S*, indicated by the figure in the top right corner of the plot in the first column. Each column corresponds to a different value of *k*: 0 on the left, with black curves, 1 in the centre, with blue curves and 2 on the right, with red curves. The plot in the top left panel with yellow background is the reference time series $r_{0}^{\left[0\right]}=r$. Under each panel, the corresponding values are reported for *P*(*s*, *k*) (italic) and *D*(*s*, *k*) (boldface).

**Example $E_{2}$** In what follows, a synthetic example $E_{2}$ is used to highlight the difference between DTW and PCC for increasing time shift, with and without a moderate noise level. This example mimics a common situation in omics data, when the activation of a gene induces a delayed activation of an inactive gene, with a similar expression level curve, affected by a certain amount of noise. Consider the following time series with 100 time points {*t*_*i*_ = *i* : 1 ≤ *i* ≤ 100}:

$$\begin{array}{c} r\left(i\right)=\frac{1}{10}e^{-\frac{2}{25}i}\sqrt{i^{3}}\sin\left(\frac{3}{20}i\right), \end{array}$$

whose graph is displayed in the top-left panel (yellow background) of Fig 2. Moreover, define the following family of time series originated by *r*(*i*), for ε(z)∈U(-z,z):

$$\begin{array}{c} r_{s}^{\left[k\right]}\left(i\right)=\{\begin{array}{cc} \varepsilon (k) & \text{for}\;i\leq s\\ r(i-s)+\varepsilon (k\cdot r(i-s)) & \text{for}\;s<i\leq 100. \end{array} \end{array}$$

In this notation, $r_{0}^{\left[0\right]}\left(i\right)=r\left(i\right)$. Finally, define the two functions

$$\begin{array}{cc} P:N\times R_{0}^{+}\to \left[-1,1\right] & D:N\times R_{0}^{+}\to \left[0,1\right]\\ \left(s,k\right)\mapsto \text{PCC}\left(r_{s}^{\left[k\right]},r\right) & \left(s,k\right)\mapsto {\text{DTW}}_{s}\left(r_{s}^{\left[k\right]},r\right) \end{array}$$

**Fig 3:**
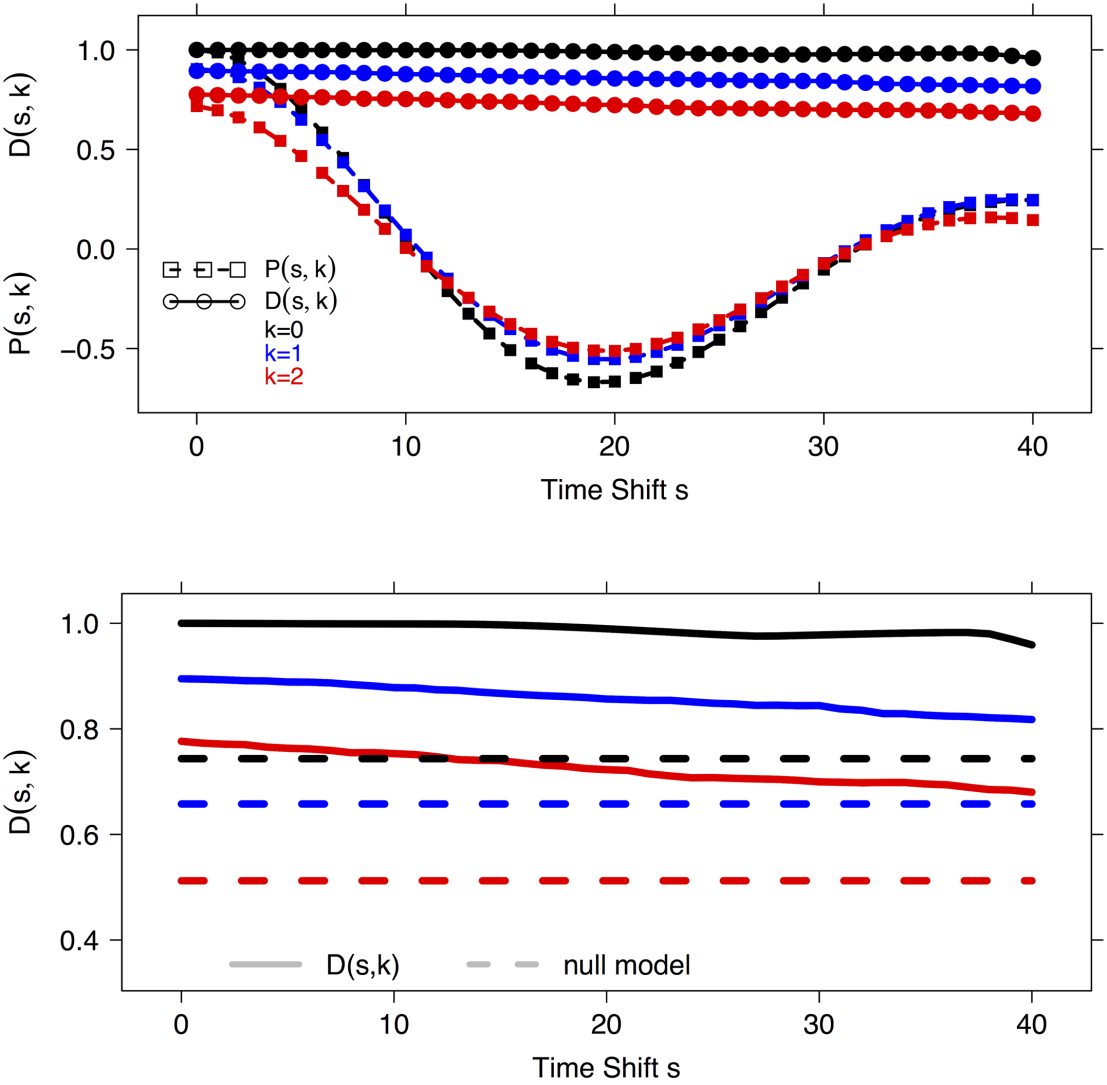
Example $E_{2}$. PCC and DTW_*s*_ versus the reference series *r* for the $\left\{r_{s}^{\left[k\right]}\right\}$ with *k* = 0, 1, 2 and the time shift *s* ranging between 0 and 40. Squares correspond to *P*(*s*, *k*), while circles and solid lines indicate *D*(*s*, *k*); the different noise levels *k* = 0, 1, 2 are denoted by curves in black, blue and red respectively. The dashed lines in the bottom panel indicate the no-information value for DTW_*s*_ based on the null model described in Example $E_{2}$.

In Fig 2 the plots of the 15 time series $\left\{r_{s}^{\left[k\right]}\left(i\right):s=0,5,10,20,40,k=0,1,2\right\}$ are shown, together with the corresponding values of *P*(*s*, *k*) (italic) and *D*(*s*, *k*) (boldface). Moreover, in the top panel of Fig 3 the curves *P*(*s*, *k*) (squares) and *D*(*s*, *k*) (dots) are displayed for *k* = 0, 1, 2 (in black, blue and red respectively) versus the time shift *s* ranging from 0 to 40. The example shows that DTW can model the dependence between $r_{s}^{\left[k\right]}\left(i\right)$ and *r*(*i*), even for large time shift *s* and high noise level *k*. In particular, as a function of the time shift *s*, the value for DTW monotonically decreases from 1 to 0.959, 0.804, 0.670 for *k* = 0, 1, 2 respectively, and *D*(*s*, 0) > *D*(*s*, 1) > *D*(*s*, 2) consistently along the whole range 0 ≤ *s* ≤ 40. On the other hand, PCC rapidly decreases to very low correlation level even for small time shifts *s* > 5, with PCC < 0.3 for all values *s* > 7. Furthermore, the PCC value does not change monotonically on increasing noise: in fact, the curves *P*(*s*, *k*) mutually intersecate. Finally, to assess the significance of the values *D*(*s*, *k*), we compare it against the null distribution $D_{m}^{M}=\left\{{\text{DTW}}_{s}\left(\eta_{j},\eta_{j+N}\right)\right\}$, where the set $\left\{\eta_{j}:\eta_{j}\in {\left[m,M\right]}^{100},1\leq j\leq 2N,\eta_{j}\left(i\right)\in U\left(m,M\right),1\leq i\leq 100\right\}$ consists of 2*N* random vectors *η*_*j*_ on 100 time points with values randomly and uniformly sampled between two positive real values *m* < *M*. In particular, as parameters here we use *N* = 1000 and, given a noise level *k*, we set $m=\min_{\begin{array}{c} 0\leq s\leq 40\\ 1\leq i\leq 100 \end{array}}r_{s}^{\left[k\right]}\left(i\right)$ and $M=\max_{\begin{array}{c} 0\leq s\leq 40\\ 1\leq i\leq 100 \end{array}}r_{s}^{\left[k\right]}\left(i\right)$. For all the three cases *k* = 0, 1, 2, the distribution of the set $D_{m}^{M}$ is Gaussian shaped, and the 95% Student bootstrap confidence intervals around the mean are quite narrow, namely (0.7429, 0.7441), (0.6570, 0.6584) and (0.5115, 0.5130) for *k* = 0, 1, 2 respectively. Thus the mean values $\bar{D_{m}^{M}}$, *i.e.*, 0.7435 (*k* = 0), 0.6577 (*k* = 1) and 0.5121 (*k* = 2), can be used as significance thresholds, as shown in the bottom panel of Fig 3: in all the three cases, for the whole range 0 ≤ *s* ≤ 40, the curve *P*(*s*, *k*) lies above the corresponding significance threshold value.

#### DTW-MIC

We define DTW-MIC as a novel measure of similarity between two time series by considering the root mean square of MIC and DTW_*s*_:

$$\begin{array}{c} \text{DTW}-\text{MIC}\left(T_{1},T_{2}\right)=\frac{1}{\sqrt{2}}\sqrt{{\text{DTW}}_{s}{\left(T_{1},T_{2}\right)}^{2}+\text{MIC}{\left(T_{1},T_{2}\right)}^{2}}. \end{array}$$

By definition, DTW-MIC joins the contributions of both MIC and DTW_*s*_, thus taking care of time shifts and non-linear functional relations. This characteristic makes DTW-MIC more effective than PCC, but also of MIC and DTW considered separately, as demonstrated by the following example $E_{3}$ on synthetic data.

**Example $E_{3}$** Consider a set **g** of three genes *g*_1_, *g*_2_ and *g*_3_ and the corresponding time series of expression $G=\{G_{1},G_{2},G_{3}\}$ on 100 time points 1 ≤ *i* ≤ 100 defined as follows:

$$\begin{array}{ccc} G_{1}\left(i\right) & = & \left\{ \begin{array}{cc} 2 & \text{for}\;1\leq i\leq 30\\ 2+\frac{i-30}{20}\sin\frac{(i-30)\pi }{70} & \text{for}\;31\leq i\leq 100 \end{array}\right.\\ G_{2}\left(i\right) & = & 3+2\sin\frac{i\pi }{100}\\ G_{3}\left(i\right) & = & 2+\log\sqrt{|\frac{1}{10}+\sin3\left(G_{2}\left(i\right)-3\right)|}. \end{array}$$

**Fig 4:**
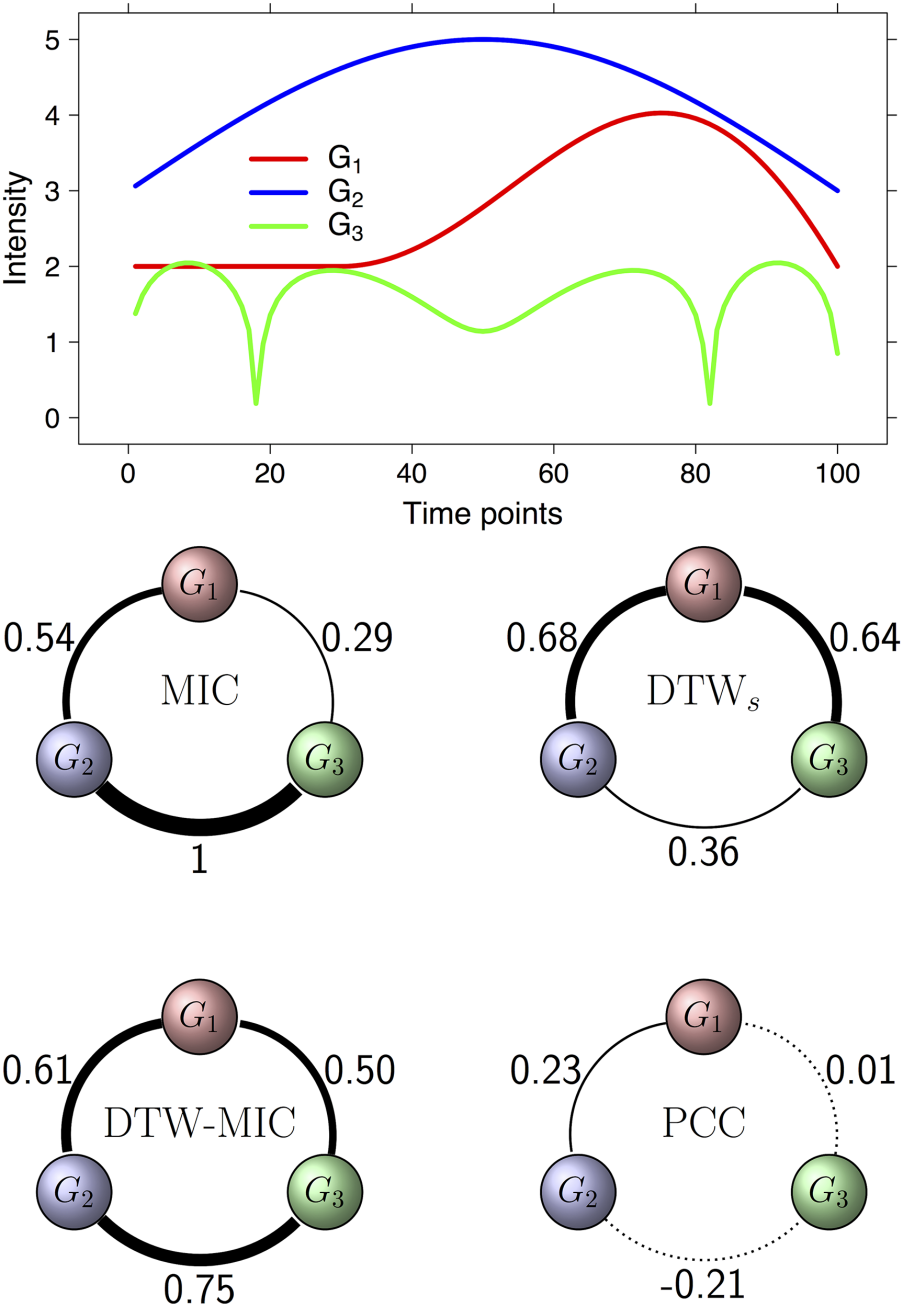
Example $E_{3}$. Plots (top) and PCC, MIC, DTW_*s*_ and DTW-MIC weighted coexpression networks (bottom) for the set G of the three time series *G*_1_, *G*_2_ and *G*_3_ (in red, blue and green respectively). Arc width is proportional to edge weight.

The graphs of the functions in G are plotted in the top panel of Fig 4, while in the bottom panel we list all values {*M*(*i*, *j*) : *M* ∈ {PCC, MIC, DTW_*s*_, DTW-MIC} and 1 ≤ *i* < *j* ≤ 3}, computing for each similarity measure *M*, the corresponding coexpression network on the gene set **g**. All the three pairs of series have a very low correlation (PCC ≤ 0.23), but DTW-MIC is still able to capture the existing relation between them (DTW-MIC ≤ 0.5), even when these relations are of different nature. In fact, *G*_2_ and *G*_3_ have a low DTW similarity, but a high MIC correlation, while the opposite happens for *G*_1_ and *G*_3_. Finally, the pair (*G*_1_, *G*_2_) has moderate values for both MIC and DTW. In all three cases the resulting DTW-MIC value is above the significance threshold computed from the null model described in the previous section, which is 0.52 for (*G*_1_, *G*_2_), 0.29 for (*G*_2_, *G*_3_) and 0.39 for (*G*_1_, *G*_3_).

### Network Analysis

#### Co-expression networks

An effective method for simultaneously analysing the mutual relations among a group of interacting agents is provided by graph theory, consisting in (i) building a complex network that has the agents as nodes and (ii) inferring the (weight of the) edges connecting the nodes by applying a similarity measure between the signals of the agents. A typical example in omics science is represented by gene networks: the nodes are the genes and an edge between two genes is weighted by the similarity between their expression levels in a time window as read by microarray or sequencing technologies. In case of a binary network, the edge is declared to exist only if the similarity value lies above a chosen threshold. These graphs are called coexpression networks, having as most popular model the Weighted Gene Co-expression Network Analysis (WGCNA) [16–18], where the adopted similarity is the absolute PCC, soft thresholded by a power function. In detail, given *N* genes and their expressions *g*_1_, …, *g*_*n*_, the resulting WGCNA network is described by the adjacency matrix *A* whose entries are defined as

$$\begin{array}{c} a_{ij}=M{\left(g_{i},g_{j}\right)}^{\beta }, \end{array}$$ (1)

for *M* = |PCC| and *β* a positive power, usually tuned according to additional constraints, such as the scale-freeness [115, 116] of the network; the default choice in the WGCNA R/Bioconductor package [17] is *β* = 6.

#### Comparison methods

In the Results section we will use the WGNCA framework with the novel DTW-MIC as the *M* measure in Eq (1), comparing the obtained networks with those inferred by the classical choice *M* = |PCC|. Apart from WGCNA, we will use two more algorithms for comparison purposes to DTW-MIC.

Algorithm for the Reconstruction of Accurate Cellular Networks (ARACNE) [42] is the reference method implementing reconstruction of network based on estimation of MI, together with Context Likelihood of Relatedness (CLR) [82], Maximum Relevance/Minimum Redundancy (MRNET) [117] and Relevance Network (RELNET) [118]. Although challenged in performance by novel MI approaches [80, 119–121], ARACNE still remains a valuable baseline when assessing the effectiveness of a novel method, and, for instance, tends to outperform approaches based on partial correlation [4, 95, 122]. However, as declared by the authors, the aim of ARACNE is the detection of transcriptional interactions with high confidence rather than the inference of all transcriptional interactions in a genetic network. Moreover, its potential goes beyond the coexpression assessment, making it suitable to address a wider range of network deconvolution problems. Since ARACNE is not designed to work on longitudinal data, as comparison method we select Time-Delay ARACNE [65], which allows the application of the ARACNE algorithm to time-course expression profiles. In detail, time-delayed dependencies between profiles are assessed by assuming a stationary Markov Random Field as the underlying probabilistic model, and then extracting a MI measure of dependence between the two genes at different time delay. Operatively, we will use Time-Delay ARACNE in its R/Bioconductor implementation provided by the *TD-ARACNE* package.

The second algorithm, Transfer Entropy [63, 64], although still based on MI estimation, has a different origin: it comes from statistical physics and it is aimed at quantifying the statistical coherence between systems evolving in time. In detail, this alternative information theoretic measure integrates the MI properties with the dynamics of information transport expressed in terms of Kullback entropy. Unlike DTW and TimeDelay-ARACNE, Transfer Entropy does not introduce any time delay in the observation, but rather it generalizes the entropy rate to two signals by measuring the deviation from independence. Hereafter we will test this measure as implemented in the *TransferEntropy* R package within the WGCNA framework with *β* = 6. Since Transfer Entropy is not symmetric, we follow the same strategy adopted by the authors of MIC: the weight of an unsigned interaction between the signals of two genes *X*, *Y* is the maximum of the intensity of the two directed interactions *X* → *Y*, *Y* → *X*. The embedding dimension and the neighbor used by the Kraskov estimator are set to 3 and 1, respectively, as shown in the documentation of the R package. In some cases, the considered dataset does not satisfy the assumptions of the Kraskov estimator, thus Transfer Entropy cannot be computed. As suggested by the R package documentation, a small Gaussian noise needs to be added to the data before computing Transfer Entropy.

#### Hamming-Ipsen-Mikhailov distance

For the quantitative assessment of the difference between two networks sharing the same nodes a graph distance is required. Among all metrics described in the literature, we choose the Hamming-Ipsen-Mikhailov (HIM) distance for its consistency and robustness [59, 123]. The HIM distance for network comparison is defined as the product metric of the Hamming distance H [124, 125] and the Ipsen-Mikhailov distance IM [126], normalized by the factor $\sqrt{2}$ to set its upper bound to 1:

$$\begin{array}{c} \text{HIM}\left(N_{1},N_{2}\right)=\frac{1}{\sqrt{2}}\sqrt{\text{H}{\left(N_{1},N_{2}\right)}^{2}+\text{IM}{\left(N_{1},N_{2}\right)}^{2}}, \end{array}$$

for *N*_1_, *N*_2_ two undirected (possibly weighted) networks. The drawback of edit distances (such as H) is their locality, as they focus only on the network parts that are different in terms of presence or absence of matching links [123]. Spectral distances like IM are global, since they take into account the whole graph structure, but they cannot distinguish isomorphic or isospectral graphs, which can correspond to quite different conditions within the biological context. The HIM distance is a solution tackling both issues: details on HIM and its two components H and IM together with a few application examples are given in [58, 59]. In particular, HIM distance can be computed also for directed networks by using an alternative description of the graph topology. Values of HIM distance range from 0 (when comparing identical networks) to 1, attained only when comparing the full and the empty network.

**Fig 5:**
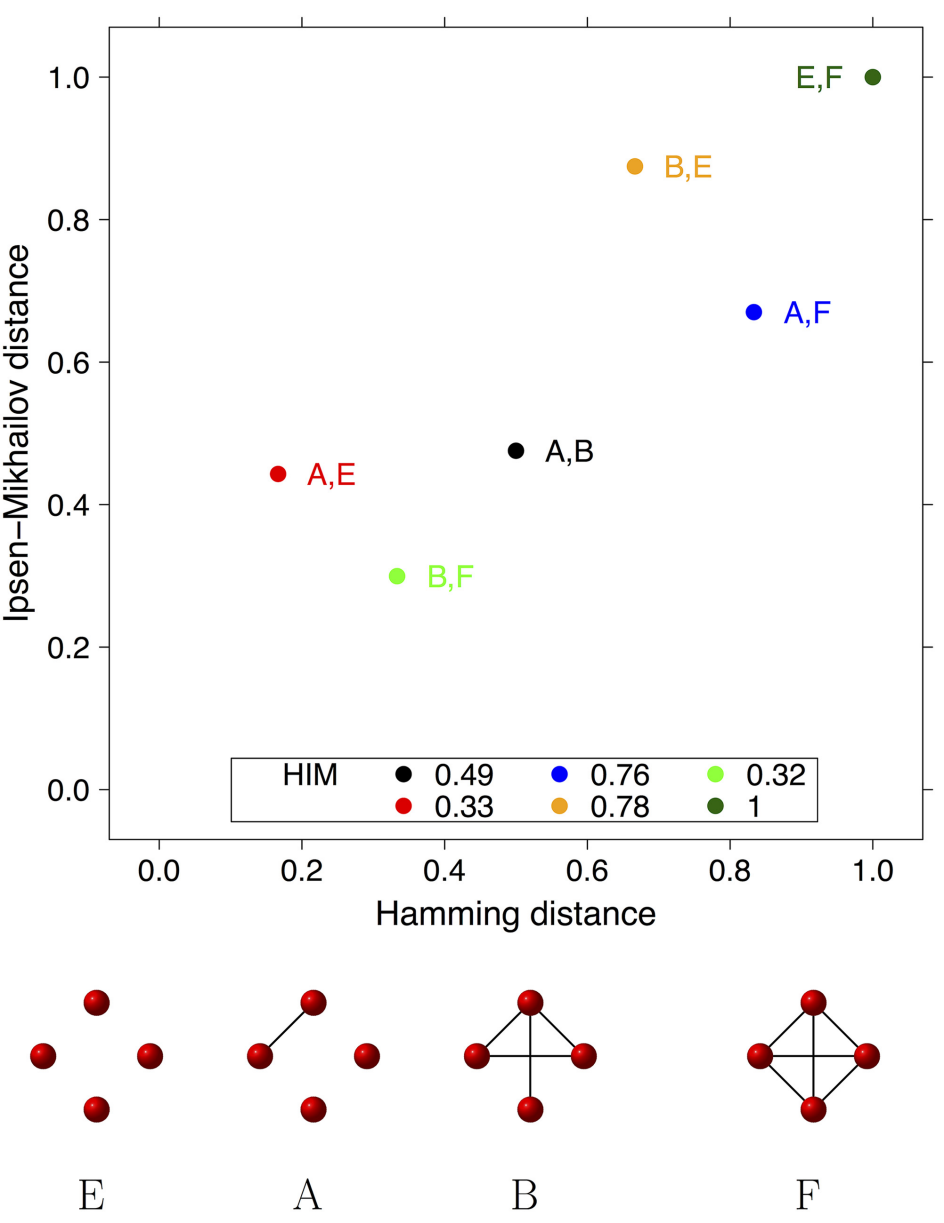
Example $E_{4}$. Mutual HIM distances in the H×IM space between 4 non-isospectral graphs A, B, E, F on 4 vertices, whose topology is shown below the plot. Distance values are listed in the plot legend.

**Example $E_{4}$** In the example $E_{4}$ shown in Fig 5, we selected four non-isospectral networks on four vertices, namely the empty graph E, the full graph F, a network with 1 edge A and a network with 4 edges including a 3-cycle, B. For these 4 graphs, the mutual H, IM and HIM distances are computed and reported as points on the H × IM plane, where each distance HIM(*P*, *Q*) between two graphs *P* and *Q* is represented by a point of coordinates *R* = (H(*P*, *Q*), IM(*P*, *Q*)) and its HIM value is the length of the segment connecting *R* to the origin (0, 0), divided by $\sqrt{2}$. The visualization in the H × IM plane allows the relative comparison of the values of the two components of the distance: for instance, the Hamming distance between A and E is half the Hamming distance between B and F (1/6 vs. 1/3), but the IM component is much larger for the former pair, yielding two quite similar values for HIM.

## Results

In this section we apply the novel DTW-MIC similarity measure to two case studies in computational biology.

Each dataset includes a network N of connections between *n* genes, together with the corresponding time series describing, for each gene, the dynamics of the expression level. Our strategy is the same in both applications and it includes two steps: first, the reconstruction of the network in the WGCNA framework in the classical approach via PCC and through DTW-MIC and the two additional benchmark measures TimeDelay ARACNE and Transfer Entropy, and then the evaluation of the HIM distance of the reconstructed networks from the true graph.

In detail, in the first application a suite of three synthetic gene network/time-course datasets is generated, inspired by real biological systems. The second task has the same goal, but expression level measurements come from a publicly available microarray dataset from a human cohort and the true network is experimentally unknown; however, a reasonable approximation of the network has been inferred by GeneNet [127, 128], a dynamical estimator of partial correlation coupled with an *ad hoc* procedure for the control of the local false discovery rate at a given threshold, an algorithm proven to be well performing in reconstruction [4]. Although the true network is not biologically validated, some landmark publications have used these datasets (*e.g.*, [129, 130]). Indeed, in the few case where an experimental validation is available, either the data are not longitudinal (*e.g.* [131]) or the time series is too short (*e.g.* [132]) to guarantee statistical significance to the MIC measure [49, 53], or the network structure is not suitable for being reconstructed by correlation-based methods, as in the cases of causal analysis or encoding directional information [9] (*e.g.* [131]).

### GeneNetWeaver Yeast & *E. coli* data

The datasets for the synthetic example are generated by GeneNetWeaver (GNW) [60, 133] an open-source tool for in silico benchmark generation, available at the web address http://gnw.sourceforge.net/genenetweaver.html. GNW generates realistic network structures of biologically plausible benchmarks by extracting modules from known gene networks of model organisms like yeast and E. coli [134], endowing them with dynamics using a kinetic thermodynamical model of transcriptional regulation with added internal noise, allowing for different types of customizable perturbations. According to the user prescribed constraints and given a chosen network topology, GNW can also produce steady states and time course datasets with the expression levels of the network nodes. The annual Dialogue for Reverse Engineering Assessments and Methods (DREAM, http://www.the-dream-project.org/) Challenge [2, 35, 61, 135–137] initiative for the quantitative comparison of network inference methods relies on GNW for the synthetic benchmark datasets.

Three synthetic networks are generated by GNW for the first application task, namely Yeast_20_, Ecoli_20_, Ecoli_50_, where the name points to the original reference network and the subscript indicates the number of nodes. In detail, Yeast_20_ is a subnet of the Yeast transcriptional regulatory network with 4441 nodes and 12873 edges [134, 138], while Ecoli_20_ and Ecoli_50_ are subnets of the *E. coli* transcriptional regulatory network with 1502 nodes and 3587 edges, corresponding to the TF-gene interactions of RegulonDB release 6.7 of May 2010 [134, 139]. In all cases, the selected genes are randomly extracted from the whole set of nodes only requiring that half of the selected nodes be regulators.

**Fig 6:**
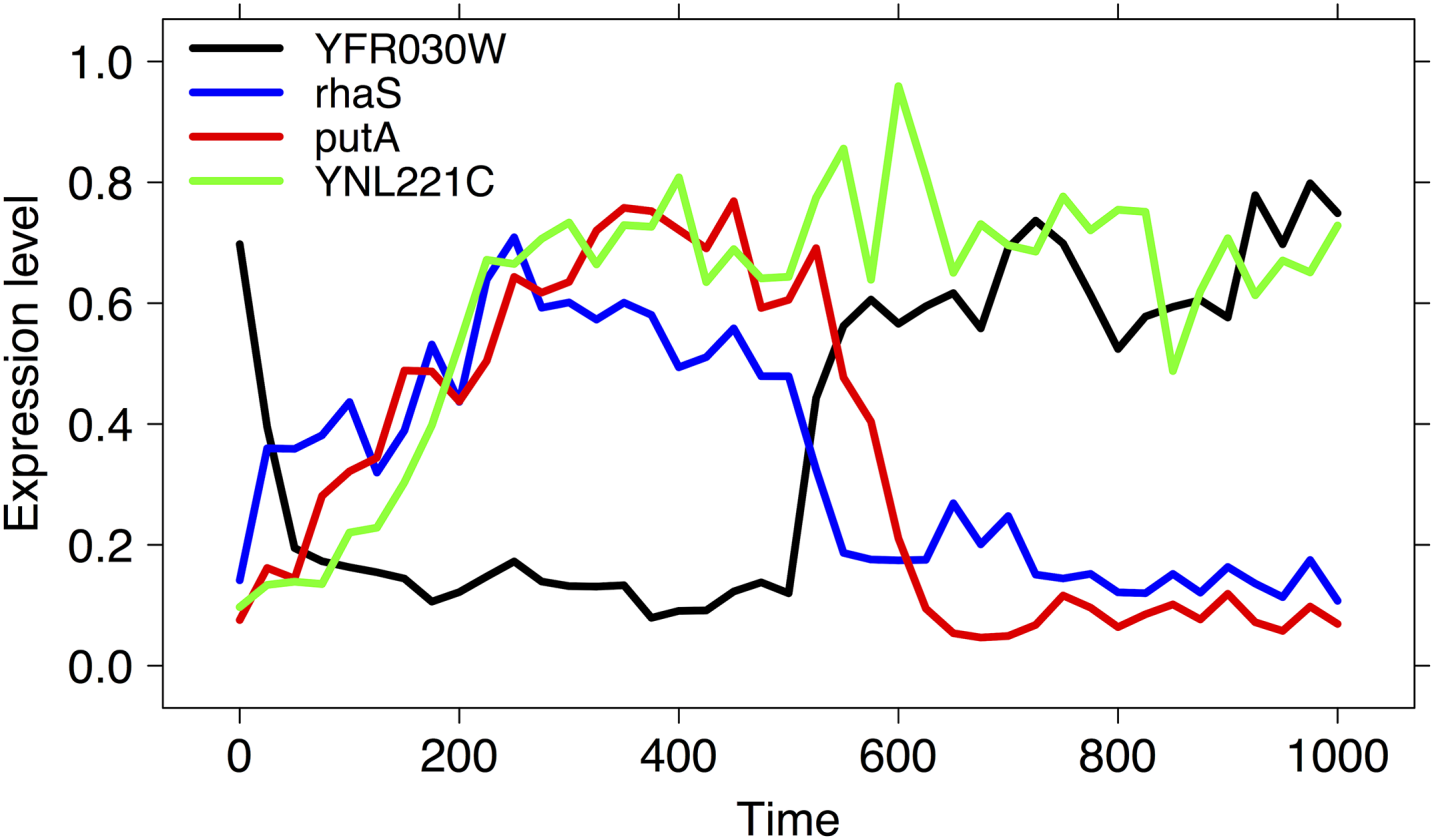
GeneNetWeaver time series. examples of 4 longitudinal expression level data generated by the GNW kinetic model for the synthetic subgraph of Yeast and *E. coli* regulatory networks. Time course data are defined on 41 time points 0, …, 1000 and they correspond to the genes YFR030W (black, from Yeast_20_), YNL221C (green, from Yeast_20_ with dual gene knockout), rhaS (from Ecoli_20_) and putA (from Ecoli_50_).

For each network, 10 longitudinal datasets {*d*_1_, …, *d*_10_} of expression levels are generated by a dynamic model mixing ordinary and stochastic differential equations, on 41 time points equally spaced between time 0 and time 1000 {*t*_0_ = 0, *t*_1_ = 25, …, *t*_40_ = 1000}. In each series, the initial time point *t*_0_ = 0 corresponds to the wild-type steady-state and, from that moment onwards, a perturbation is applied until time point *t*_20_ = 500: at that point, the perturbation is removed, and the gene expression level goes back from the perturbed to the wild-type state [134]. Moreover, a moderate level of noise is added to all the datasets, namely 0.5% for the Yeast data and 1% for the *E.coli* data; in both cases, the selected model is the microarray noise model described in [140]. Both the noise model and the perturbation scheme are chosen according to the configuration of the DREAM4 challenge [134]. As an example, in Fig 6 we show the plots of the generated time course data of four genes belonging to the selected subnets Yeast_20_, Ecoli_20_ and Ecoli_50_. GNW network and time-course data are publicly available on figshare, at the URL https://figshare.com/articles/Gene_Net_Weaver_Dataset/2279628.

**Fig 7:**
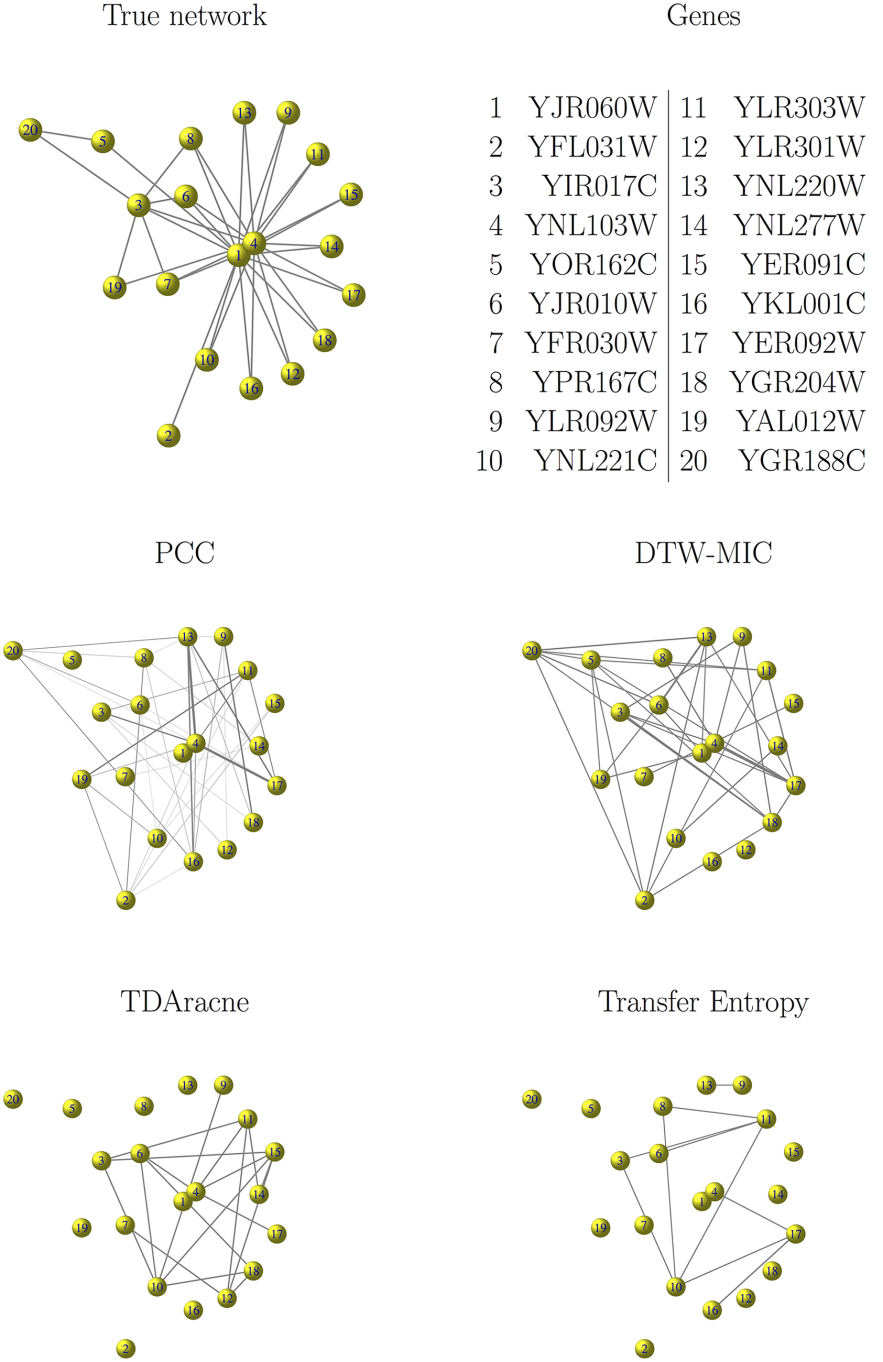
GeneNetWeaver data. example of network reconstruction and comparison with the true network. In the top panels, the topology of the synthetic true network Yeast_20_ (top left) is shown together with the Systematic Name of its 20 genes (top right). In the two bottom panels, the network Yeast_20_ as inferred from the time course dataset *d*_1_ by PCC (middle left), DTW-MIC (middle right), TimeDelay ARACNE (bottom left) and Transfer Entropy (bottom right). For the reconstructed networks, edge width is proportional to arc weight; edges with smaller weights (threshold is 0.001 for PCC, 0.135 for DTW-MIC and 0.005 for Transfer Entropy) are not drawn to avoid cluttering the image. Distance from the true network is 0.57 for the inference by PCC, 0.22 for the reconstruction by DTW-MIC, 0.28 for TimeDelay ARACNE and 0.57 for Transfer Entropy.

In each of the three cases Yeast_20_, Ecoli_20_ and Ecoli_50_, a network is inferred by PCC, DTW-MIC, Transfer Entropy and TimeDelay ARACNE from each of the time course dataset {*d*_1_, …, *d*_10_}, and the obtained graph is compared via the HIM distance to the corresponding true network. As an example, in Fig 7 we show the true Yeast_20_ graph aside the networks reconstructed from the dataset *d*_1_. In all experiments, the results for TimeDelay ARACNE are reported for *N* = 11 normalization bins and likelihood 1.2 as in the R package documentation; worse results (not reported here) were obtained for *N* = 5 and *N* = 22.

**Fig 8:**
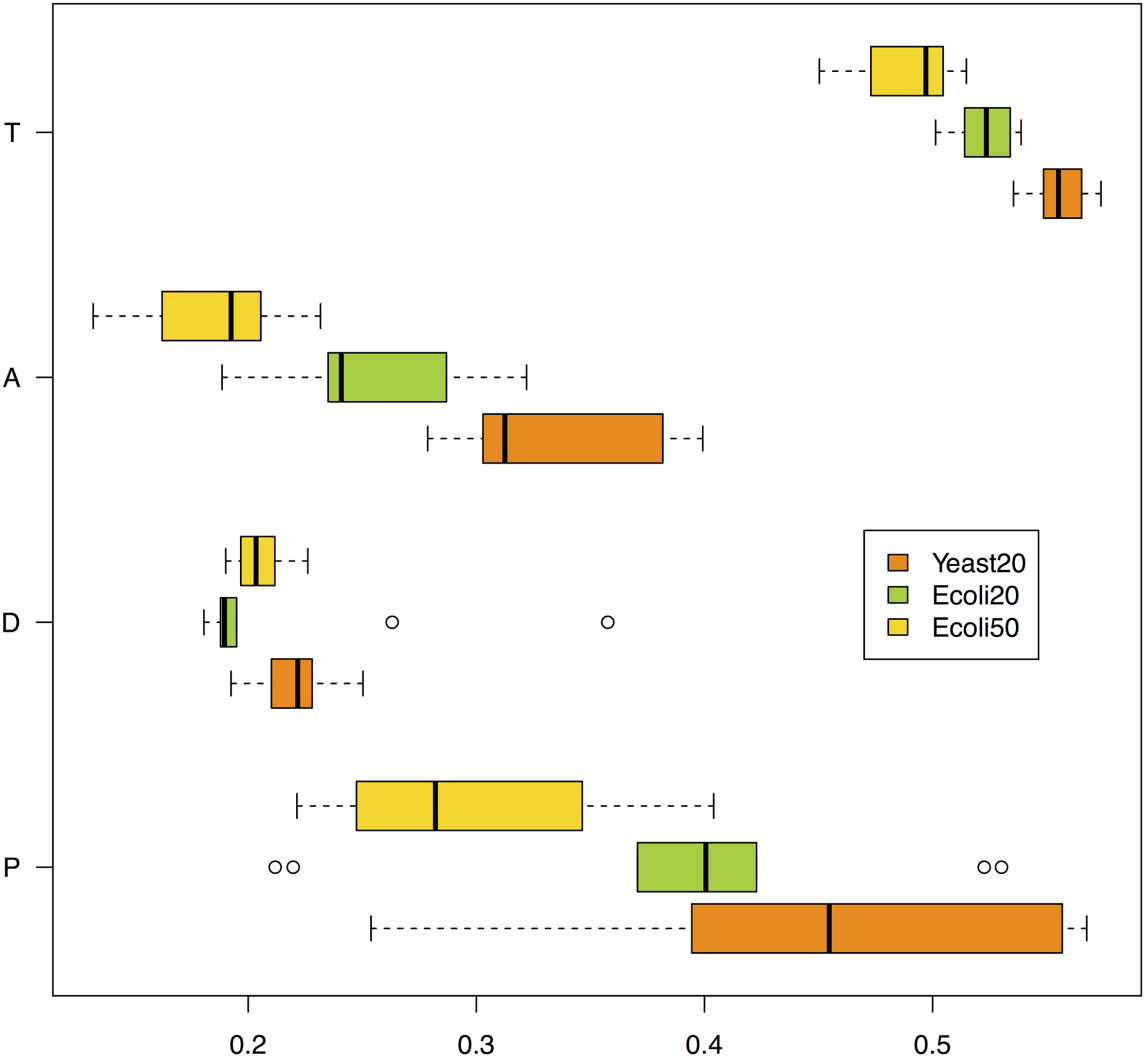
GeneNetWeaver data. box and whisker plot of the HIM distance between the networks inferred from time series and the true graphs, listed in Table 1. For each true network Yeast_20_, Ecoli_20_ and Ecoli_50_, 10 different graphs are reconstructed by PCC, DTW-MIC, TimeDelay ARACNE and Transfer Entropy similarity measures.

The results are reported in Table 1 and summarized in the box and whisker plots of Fig 8. The networks inferred by DTW-MIC are consistently closer to the true network than the graphs created with other inference methods, apart from Ecoli_50_ with TimeDelay ARACNE, with also smaller standard deviation over the 10 experiments in almost all cases.

**Table 1:** HIM distances with basic statistics of the DTW-MIC (D), the PCC (P), the Transfer Entropy (T) and the Time-Delay ARACNE (A) inferred networks for all experiments on the GNW datasets Yeast_20_, Ecoli_20_, Ecoli_50_.

# Dataset	Yeast_20_				Ecoli_20_				Ecoli_50_			
	P	D	A	T	P	D	A	T	P	D	A	T
*d*_1_	0.57	0.22	0.28	0.57	0.37	0.19	0.23	0.52	0.22	0.21	0.23	0.47
*d*_2_	0.41	0.21	0.38	0.55	0.41	0.18	0.24	0.50	0.31	0.19	0.16	0.50
*d*_3_	0.39	0.20	0.31	0.57	0.38	0.19	0.25	0.52	0.23	0.23	0.23	0.45
*d*_4_	0.25	0.25	0.30	0.55	0.22	0.36	0.29	0.53	0.27	0.21	0.19	0.51
*d*_5_	0.56	0.24	0.39	0.57	0.41	0.19	0.32	0.52	0.35	0.19	0.19	0.51
*d*_6_	0.35	0.19	0.36	0.55	0.53	0.19	0.19	0.54	0.40	0.20	0.13	0.50
*d*_7_	0.56	0.23	0.32	0.56	0.39	0.19	0.24	0.51	0.26	0.20	0.17	0.50
*d*_8_	0.42	0.22	0.40	0.55	0.52	0.19	0.24	0.53	0.29	0.21	0.21	0.46
*d*_9_	0.49	0.22	0.30	0.56	0.42	0.19	0.24	0.53	0.25	0.21	0.19	0.47
*d*_10_	0.53	0.22	0.30	0.54	0.21	0.26	0.32	0.50	0.35	0.20	0.14	0.50
Mean	0.45	0.22	0.33	0.56	0.39	0.21	0.25	0.52	0.29	0.20	0.18	0.49
Median	0.45	0.22	0.31	0.56	0.40	0.19	0.24	0.52	0.28	0.20	0.19	0.50
Std. Dev.	0.10	0.02	0.04	0.01	0.11	0.06	0.04	0.01	0.06	0.01	0.03	0.02

For the Yeast_20_ dataset, four additional time course datasets were generated on the same timepoints, but with a dual gene knockout: the curve of gene YNL221C in Fig 6 is an example of the generated trajectories. The results of the HIM distances from the true network for the networks inferred by the PCC, DTW-MIC, Transfer Entropy and TimeDelay ARACNE on the four datasets *d*_1_, …, *d*_4_ are reported in Table 2.

**Table 2:** HIM distance from the true network TN Yeast_20_ of the networks inferred on the knock-out series *d*_1_, …, *d*_4_ by the four algorithm PCC, DTW-MIC, Transfer Entropy and TimeDelay ARACNE.

	*d*_1_	*d*_2_	*d*_3_	*d*_4_
DTW-MIC	0.23	0.21	0.24	0.21
PCC	0.30	0.27	0.31	0.47
TimeDelay ARACNE	0.24	0.28	0.28	0.26
Transfer Entropy*	0.29	0.28	0.31	0.24

* *d*_1_, …, *d*_4_ do not satisfy the assumptions of the Kraskov estimator, thus Transfer Entropy cannot be computed. As suggested by the R package documentation, a small Gaussian noise $\varepsilon \in N(\mu =0,\sigma =\sqrt{0.05*\sigma^{2}(d_{i}})$ was added to *d*_*i*_ before computing Transfer Entropy.

Again, the DTW-MIC inferred networks are closer to the true network than the other graphs, in all four experiments, with TimeDelay ARACNE as second best performing algorithm.

### Human T-cell data

**Fig 9:**
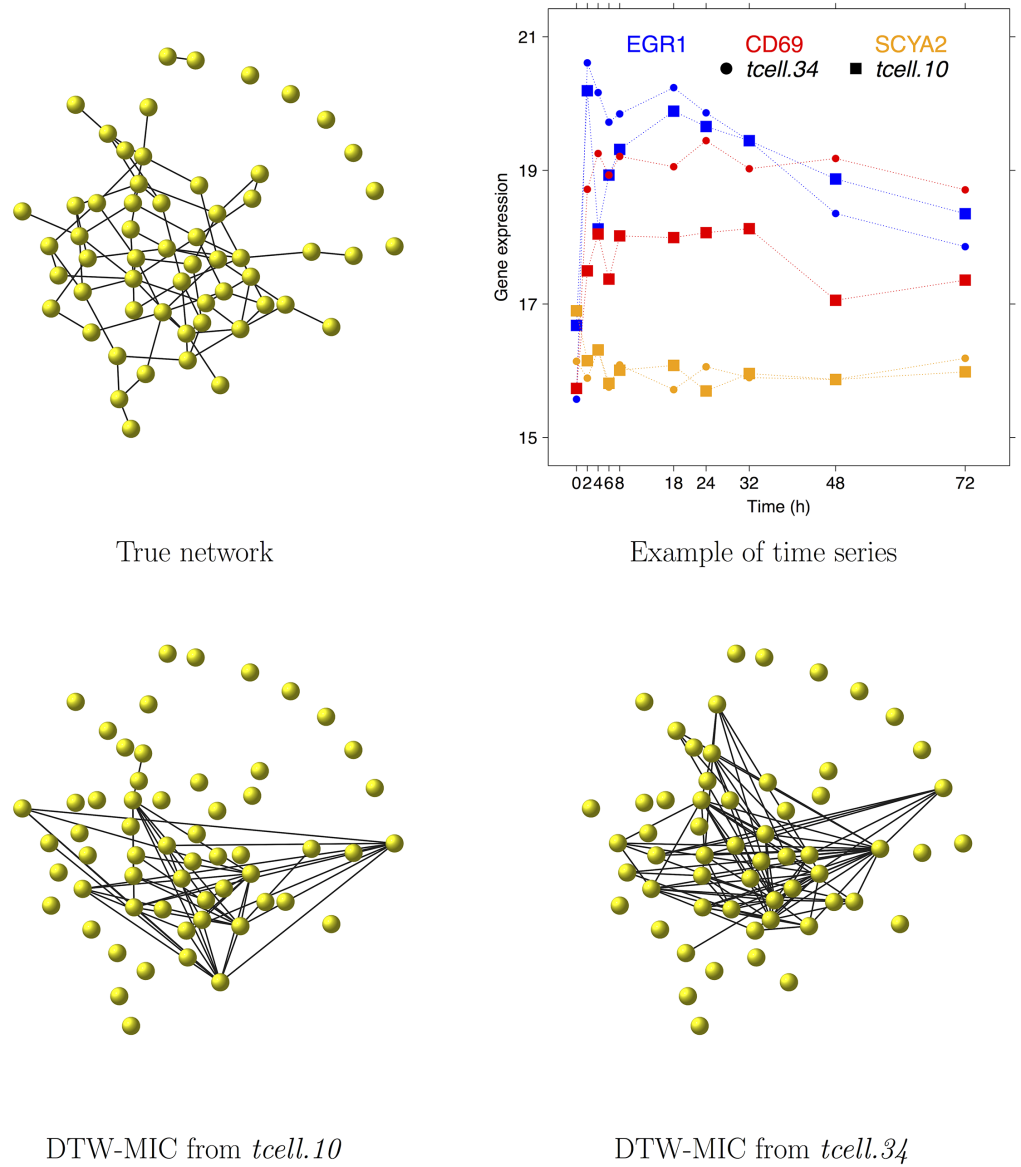
The T-cell example: True and DTW-MIC network. The (true) network as reconstructed by Opgen-Rhein and Strimmer [128] (top left); the time course for three example genes EGR1 (blue), CD69 (red) and SCYA2 (orange), from replicate 1 of the *tcell.34* (circles) and of the *tcell.10* (squares) dataset. In the second row, the networks inferred by DTW-MIC from the *tcell.10* (left) and from the *tcell.34* (right) dataset; in these last two graphs, edges with weight smaller than 0.225 are not displayed.

Rangel and colleagues in [62] investigated the dynamics of the activation of T-lymphocites by analysing the response of the human Jurkat T-cell line subjected to a treatment with phorbol 12-myristate 13-acetate (PMA) and ionomycin. Operatively, they measured the expression of 58 genes across 10 time points (0, 2, 4, 6, 8, 18, 24, 32, 48, and 72 hours after treatment) with two series of respectively 34 and 10 replicates on a custom microarray built by spotting PCR products on amino-modified glass slides using a Microgrid II spotter. The preprocessed array data *tcell.34* and *tcell.10*, log-transformed and quantile normalized, are publicly available in the R package *longitudinal*. This package was developed by Opgen-Rhein and Strimmer who inferred the corresponding network by shrinkage estimation of the (partial) dynamical correlation [128, 141]. Their result is considered here as the true network, displayed in the top left panel of Fig 9. As an example of the data in the *tcell.34* and *tcell.10*, in the top right panel of the same Fig 9 we show the time course data for the three genes EGR1, CD69 and SCYA2 in the first out of 34 replicates of *tcell.34* and in the first out of 10 replicates of *tcell.10*.

**Fig 10:**
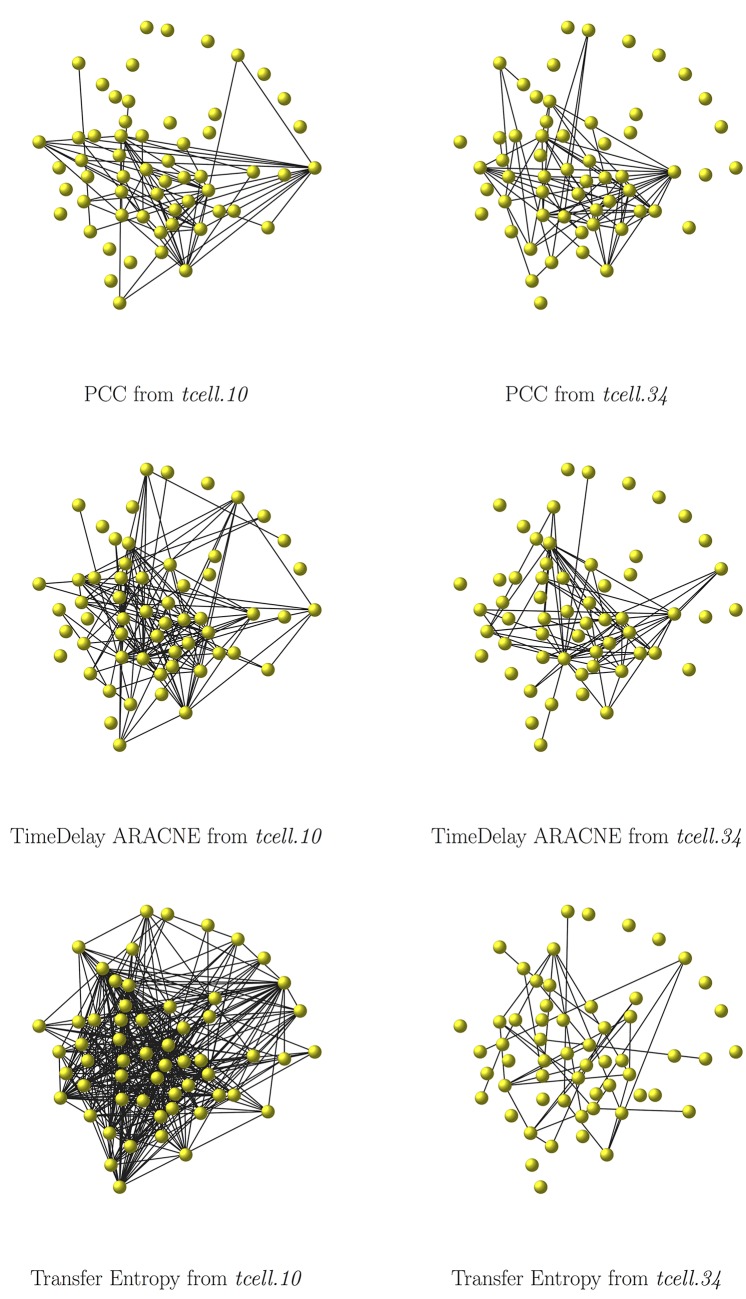
The T-cell example: comparison networks. The Human t-cell network as reconstructed by PCC (top row), TimeDelay ARACNE (middle row) and Transfer Entropy (bottom row), from the *tcell.10* (left column) and from the *tcell.34* (right column) dataset. Edges with weights smaller than 0.1 for PCC and smaller than 0.0001 for Trasfer Entropy are not displayed.

Eight instances of the T-cell network are inferred, by the three similarity measures DTW-MIC, PCC and Transfer Entropy and the reconstruction algorithm TimeDelay ARACNE, starting from the two datasets *tcell.34* and *tcell.10*. In both datasets, the dimension of the longitudinal data for each replicate (10 time points) cannot guarantee robustness in the inference process, since both PCC and MIC are not reliable for datasets of too small sample size [49, 68, 142]. Hence all replicates in the two datasets are consecutively joined so that time point 72h of replicate *i* is followed by time point 0h for replicate *i* + 1, thus yielding for each gene a single time course on 340 time points for *tcell.34* and on 100 time points for *tcell.10*. The inferred networks are displayed in Figs 9 and 10, while in Table 3 the H/IM/HIM distances are reported between the true and the inferred T-cell networks.

**Table 3:** Hamming (H, top matrix, upper triangle), Ipsen-Mikhailov (IM, top matrix, lower triangle) and HIM (bottom matrix) distances among the true (TN) and the T-cell WGCNA inferred networks, by DTW-MIC (D), PCC (P), Transfer Entropy (T) and Time-Delay ARACNE (A) similarity measure, from the *tcell.34* (34) and the *tcell.10* (10) time course datasets.

	TN	*D*_34_	*P*_34_	*T*_34_	*A*_34_	*D*_10_	*P*_10_	*T*_10_	*A*_10_
TN	◼	0.11	0.06	0.05	0.08	0.09	0.06	0.05	0.10
*D*_34_	0.20	◼	0.06	0.07	0.10	0.05	0.07	0.07	0.13
*P*_34_	0.30	0.49	◼	0.02	0.05	0.05	0.02	0.02	0.08
*T*_34_	0.70	0.85	0.49	◼	0.05	0.05	0.01	0.00	0.08
*A*_34_	0.65	0.81	0.45	0.05	◼	0.05	0.01	0.00	0.09
*D*_10_	0.18	0.17	0.43	0.83	0.35	◼	0.04	0.05	0.11
*P*_10_	0.35	0.55	0.06	0.48	0.28	0.47	◼	0.01	0.08
*T*_10_	0.68	0.84	0.45	0.07	0.49	0.81	0.44	◼	0.08
*A*_10_	0.63	0.78	0.43	0.07	0.18	0.76	0.42	0.09	◼
	*D*_34_	*P*_34_	*T*_34_	*A*_34_	*D*_10_	*P*_10_	*T*_10_	*A*_10_
TN	0.16	0.21	0.49	0.16	0.14	0.25	0.48	0.14	
	*D*_34_	0.35	0.60	0.26	0.13	0.39	0.60	0.21	
		*P*_34_	0.34	0.17	0.30	0.04	0.32	0.24	
			*T*_34_	0.36	0.59	0.34	0.05	0.48	
				*A*_34_	0.26	0.20	0.35	0.14	
					*D*_10_	0.34	0.57	0.22	
						*P*_10_	0.31	0.28	
							*T*_10_	0.47	

The two datasets *tcell.i*, *i* = 10, 34 do not satisfy the assumptions of the Kraskov estimator, thus Transfer Entropy cannot be computed. As suggested by the R package documentation, a small Gaussian noise $\varepsilon \in N(\mu =0,\sigma =\sqrt{0.05*\sigma^{2}(tcell.i})$ was added to *tcell.i*, *i* = 10, 34 before computing Transfer Entropy.

**Fig 11:**
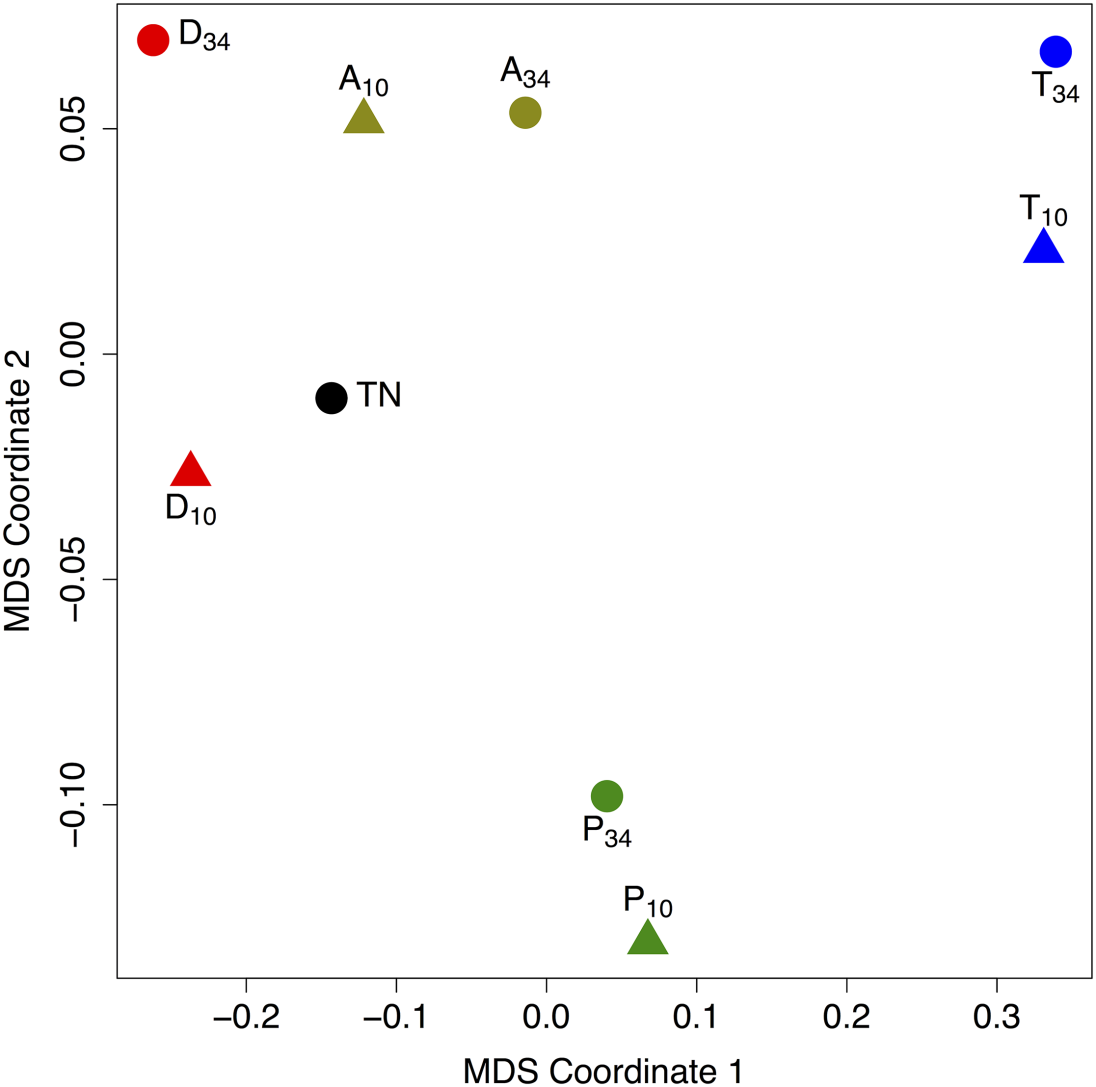
Metric multidimensional scaling of HIM distances. Planar projection conserving the mutual distances between the true Human t-cell network (TN) and the eight networks inferred from the two datasets *tcell.34* (⋅_34_) and *tcell.10* (⋅_10_) by the four reconstruction algorithms DTW-MIC (D), PCC (P), Transfer Entropy (T) and TimeDelay ARACNE (A).

In Fig 11 we show the plot of the metric multidimensional scaling of all mutual HIM distances.

For both datasets *tcell.34* and *tcell.10* the HIM distance from the true graph is smaller for the networks inferred by the DTW-MIC. The TimeDelay ARACNE measure reaches the same results, but only after a tuning phase optimizing the parameters *N* = 11, *δ* = 3 and likelihood 0.7. Note that, in all cases, the Hamming component of the distance is smaller, while the Ipsen-Mikhailov component is larger. Thus less links are changing between the inferred networks and the true graph, but these changing links induce a strongly different structure between the two nets. Indeed, in this experiment the choice of the similarity measure has a larger impact than the starting dataset, since the nets inferred using the same measure on different datasets are mutually closer than the nets inferred by different methods on the same time courses. Finally, without the power function (with *β* = 6 as default) applied in the WGCNA for soft thresholding the reconstructed networks are very different from the true graph, regardless of the starting dataset. For instance, the resulting HIM is about 0.47 for PCC and 0.66 for DTW-MIC, with 0.63 the average HIM value for a null model generated by computing the distance from the true graph of 1000 random network with uniform edge weight distribution in (0, 1). This effect does not come unexpected, because of the tendency of MIC to overestimate the MI in a number of situations [9, 22, 67, 68], thus generating false positives. An effective solution to avoid this bias and obtaining a more reliable estimate is the use of a thresholding function, either hard as in [142] or soft as in this case via a power law: these procedures allow discarding completely (hard threshold) or greatly reducing the weight of (soft threshold) the unwanted links wrongly detected by the association measure.

## Conclusions

We introduced here DTW-MIC, a novel similarity measure for inferring coexpression networks from longitudinal data as an alternative to the absolute PCC used in the WGCNA approach. By combining Dynamic Time Warping and Maximal Information Coefficient, the DTW-MIC similarity can overcome the well known limitations of PCC when dealing with delayed signals and indirect interactions. Experiments on biologically inspired synthetic data and gene expression time course data demonstrate higher precision on average in the network inference for DTW-MIC with respect to PCC, TimeDelay ARACNE and Transfer Entropy in different conditions, and without the need for a parameter tuning phase. Considering the MIC bias towards false positives and the availability of numerous similarity measures derived from DTW, it is likely to expect as future development the exploration of different alternatives to the DTW-MIC pair. For instance, it has been pointed out that Brownian distance correlation [67, 76] and biweight midcorrelation [9, 143] do not suffer from the issues affecting MIC, and thus they may be adopted as replacements for MIC; on the other hand, ComplexityInvariantDTW [109] appears to be the most effective alternative to DTW, well performing on a wide range of situations, and thus worth exploring as a potential substitute.
